# Evaluation of the immunomodulatory effects of anti-COVID-19 TCM formulae by multiple virus-related pathways

**DOI:** 10.1038/s41392-021-00475-w

**Published:** 2021-02-04

**Authors:** Liansheng Qiao, Wenting Huang, Xiaoling Zhang, Hongyan Guo, Dong Wang, Quansheng Feng, Ronghua Jin, Lan Xie, Weimin Li, Jing Cheng

**Affiliations:** 1 State Key Laboratory of Membrane Biology, School of Medicine, Tsinghua University, Beijing, 100084 China; 2 Collaborative Innovation Center for Diagnosis and Treatment of Infectious Diseases, Hangzhou, 310003 China; 3 Medical Systems Biology Research Center, School of Medicine, Tsinghua University, Beijing, 100084 China; 4 National Engineering Research Center for Beijing Biochip Technology, Beijing, 102206 China; 5 School of Basic Medical Sciences, Chengdu University of Traditional Chinese Medicine, Chengdu, 611137 China; 6 Beijing Ditan Hospital, Capital Medical University, Beijing, 100015 China; 7 Department of Respiratory and Critic Care Medicine, West China Hospital, Sichuan University, Chengdu, 610041 China

**Keywords:** Systems biology, Infectious diseases, Computational biology and bioinformatics

**Dear Editor**,

The coronavirus infectious disease 2019 (COVID-19) outbreak is seriously endangering human health. Most patients with severe COVID-19 are characterized by sustained cytokine production and hyper-inflammation, which is known as cytokine storm syndrome.^1–3^ Elaborating the anti-inflammatory response is crucial to these patients, and interleukin-6 (IL6) inhibitors and steroids have been recommended in clinical practice.^1^ However, after the cytokine storm phase, the host immune response to sepsis may develop into a protracted immunosuppressive phase. Agents enhancing host immunity administered to patients in the immunosuppressive phase of sepsis could improve survival. Therefore, different treatments should be provided to different COVID-19 patients with cytokine storm phase or immunosuppression phase.

Traditional Chinese medicine (TCM) has been widely used to treat over 90% of the patients with COVID-19 in Chinese hospitals^4^ and among all patients with mild and moderate symptoms in Wuhan, none have generated severe symptoms when they were only treated with TCM. It is essential to evaluate their efficacy and select the most appropriate ones in a specific context.

We collected 125 anti-COVID-19 TCM formulae from public sources (The formulae and their sources are summarized in Table S1) containing 196 TCMs (Table S2), 166 of which were included in our study.

To establish gene signature profiles of 166 TCMs, we performed high-throughput sequencing-based high-throughput screening (HTS^2^) on phorbol-12-myristate-13-acetate-induced THP-1 cells. A set of 3267 genes were detected and these genes were derived from 139 pathways related to virus infection, immunity, inflammation, metabolism, cell proliferation, apoptosis, and migration (Table S3). A gene set enrichment analysis (GSEA) was used to perform pathway enrichment analyses of 11 virus-related pathways in cells exposed to the 166 TCMs (Fig. S1 and Table S4). The following 11 pathways were included: human papillomavirus infection, Kaposi sarcoma-associated herpesvirus infection, human T-cell leukemia virus 1 infection, Epstein-Barr virus infection, human immunodeficiency virus 1 infection, influenza A, human cytomegalovirus infection, hepatitis C, herpes simplex virus 1 infection, hepatitis B and measles.

Most virus-related pathways involved the Toll-like receptor signaling pathway, JAK-STAT signaling pathway, NF-κB signaling pathway, RIG-I-like receptor signaling pathway, and antigen processing and presentation, suggesting an immune response after viral infection. The efficacy of each individual TCM against each virus-related pathway was represented by the normalized enrichment score (NES). A positive NES indicates that the pathway is enriched in upregulated genes, while a negative NES indicates enrichment in downregulated genes. The efficacy of each anti-COVID-19 TCM formula was calculated as the sum of the NESs of each TCM in the formula for the pathways. In our study, we evaluated the efficacy of the TCM formulae from two aspects. For patients with cytokine storm syndrome, we proposed TCM formulae with negative NESs for virus-related pathways; and for immunosuppression, we proposed TCM formulae with positive NESs.

In addition, representative transcriptome datasets related to COVID-19 were analyzed, including leukocytes, macrophages, and lung tissues from COVID-19 patients, and SARS-CoV-2-infected cell lines.^2,3^ The NESs for the 11 virus-related pathways were positive in all 7 SARS-CoV-2-infected samples (Table S5), suggesting that the immune response was induced after viral infection.

Hierarchical clustering was performed for the 132 samples (125 formulae and 7 SARS-CoV-2-infected samples) (Fig. 1a) by considering the 11 virus-related pathways, and three groups were identified. The formulae in the same group have similar effects on 11 virus-related pathways, which may be a result of similar TCM composition and they may be suitable for patients with similar symptoms. The first group contained 22 formulae and 7 SARS-CoV-2-infected samples, which had positive NESs in most pathways and may work to activate the immune response (Fig. 1a, cluster 3). There were 45.5% and 40.9% of formulae in this group containing *Citri Reticulatae Pericarpium* (Chenpi) and *Ophiopogonis Radix* (Maidong), which were characterized as health-strengthening TCM and can be used to treat impaired type-I interferon and lymphopenia. The second group contained 21 formulae with negative NESs for most pathways, indicating anti-inflammatory effects (Fig. 1a, cluster 1). Maxing Shigan Decoction was contained in 28.6% of formulae in this group. *Glycyrrhizae Radix et Rhizoma* (Gancao) and *Ephedrae Herba* (Mahuang) were in 57.1% of formulae in this group respectively, which were recognized as TCMs relieving exterior syndrome and dispelling cold and can inhibit IL6 and alleviate inflammation. The third group contained the remaining formulae, which had mixed or relatively weak regulatory effects on the pathways (Fig. 1a, cluster 2).

**Fig. 1:**
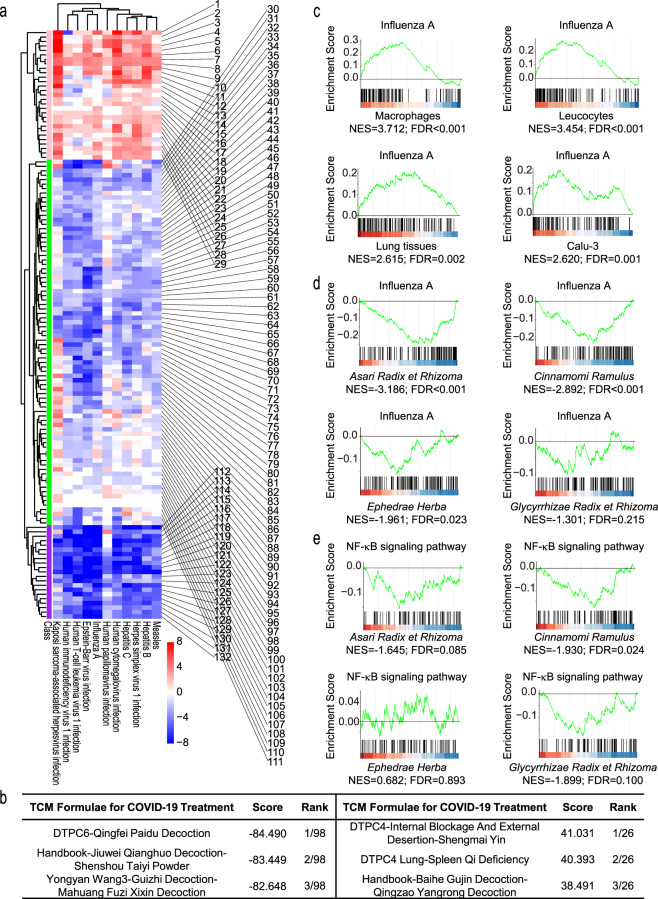
The immunomodulatory effects of the 125 anti-COVID-19 TCM formulae for virus-related pathways. **a** Heatmap of 11 virus-related pathways for 125 anti-COVID-19 TCM formulae and 7 SARS-CoV-2-infected samples. The color of each spot in the heatmap represents the NES for each pathway in each sample (false discovery rate < 0.25) along a color gradient from blue (NES < 0) to red (NES > 0). The purple bar () represents cluster 1, the green bar () represents cluster 2 and the pink bar () represents cluster 3. The order of 132 samples appearing in the heatmap is shown in Table S5. **b** Representative anti-COVID-19 formulae with negative NESs (top 3) and positive NESs (top 3) based on 11 virus-related pathways. **c** GSEA tracing for Influenza A. Influenza A pathway is upregulated in SARS-CoV-2-infected samples, including macrophages, leukocytes, lung tissues from COVID-19 patients and SARS-CoV-2-infected Calu-3 cells. **d** Influenza A pathway is downregulated in *Asari Radix et Rhizoma* (Xixin), *Cinnamomi Ramulus* (Guizhi), *Ephedrae Herba* (Mahuang) and *Glycyrrhizae Radix et Rhizoma* (Gancao) treated THP-1-derived macrophages. **e** GSEA tracing for NF-κB pathway. NF-κB signaling pathway is downregulated in *Asari Radix et Rhizoma*, *Cinnamomi Ramulus* and *Glycyrrhizae Radix et Rhizoma* treated THP-1-derived macrophages, while is not enriched in *Ephedrae Herba* treated cells

To quantitatively evaluate the efficacy of the TCM formulae, we defined the efficacy as a score by summing the NESs for each TCM formula on the 11 virus-related pathways (Table S5). The scores of all formulae ranged from −84.490 to 41.031. The scores of the SARS-CoV-2-infected samples ranged from 6.467 to 40.734. A total of 98 formulae had a negative NES, indicating their potential anti-inflammatory effects. The top 3 formulae are listed in Fig. 1b: DTPC6-Qingfei Paidu Decoction, Handbook-Jiuwei Qianghuo Decoction-Shenshou Taiyi Powder, and Yongyan Wang3-Guizhi Decoction-Mahuang Fuzi Xixin Decoction. The core components of these three formulae are *Asari Radix et Rhizoma* (Xixin), *Cinnamomi Ramulus* (Guizhi), *Ephedrae Herba*, and *Glycyrrhizae Radix et Rhizoma*. These TCMs can reverse multiple over-activated virus-related pathways of SARS-CoV-2-infected samples, for example, influenza A (Fig. 1c and d). In addition, *Asari Radix et Rhizoma*, *Cinnamomi Ramulus* and *Glycyrrhizae Radix et Rhizoma* downregulated NF-κB signaling pathway (Fig. 1e), which is a crucial pathway of cytokine storm in severe COVID-19. In contrast, 26 formulae had a positive NES for virus-related pathways, indicating an immune-activating effect. DTPC4-Internal Blockage And External Desertion-Shengmai Yin, DTPC4-Lung-Spleen Qi Deficiency, and Handbook-Baihe Gujin Decoction-Qingzao Yangrong Decoction were the top 3 formulae with the highest positive NESs (Fig. 1b). The ginsenosides isolated from Shengmai Yin have been shown to promote phagocytosis and TNF-α production by macrophages.^5^

Our research suggested a good approach for evaluating the efficacy of anti-COVID-19 TCM formulae based on their immunoregulatory effects on a macrophage model. The state council of the People’s Republic of China recommended three patented medicines and three TCM formulae for the treatment of COVID-19 which have shown good anti-COVID-19 effects in clinical practice.^4^ Our system indicated that five of six formulae and medicines mentioned above possessed a negative NES, including Qingfei Paidu Decoction (ranking 1/98), Lianhua Qingwen Capsule (ranking 6/98), Xuanfei Baidu Formula (ranking 14/98), Huashi Baidu Formula (ranking 20/98), and Jinhua Qinggan Granule (ranking 45/98). The remaining one, Xuebijing Injection (ranking 26/26), had a positive NES (Table S5). Xuebijing Injection showed a weak effect on virus-related pathways, which is probably because its major role is to attenuate microcirculation disorder and protect the endothelium from injury. However, our study is focused on the evaluation of the immunoregulatory effect of formulae. Certain TCM formulae have good antiviral activity but cannot be evaluated by our current system, and future studies are needed to explore the direct inhibitory effect of different formulae on SARS-CoV-2.

Here we established a formulae-TCM-cell-gene-pathway network, based on which 125 anti-COVID-19 TCM formulae were identified with anti-inflammatory or immune-activating functions. The rational use of the two types of formulae and their component TCMs is anticipated to provide therapeutic benefits for patients with different features of COVID-19, which can be taken into consideration by physicians both in China and all over the world.

## Supplementary information

Figure S1

Supplementary Files

Table S1-S5

## Data Availability

The signature gene expression data in this study can be found online (http://mcmm.capitalbio.com/), and the signature gene expression profiles of more TCMs in more cell lines will be updated continuously.
