# Supplementary figures and images for: Evaluation of the immunomodulatory effects of anti-COVID-19 TCM formulae by multiple virus-related pathways
Source: Signal Transduct Target Ther. 2021 Feb 4;6:50. doi: 10.1038/s41392-021-00475-w (PMC7860167; doi:10.1038/s41392-021-00475-w)

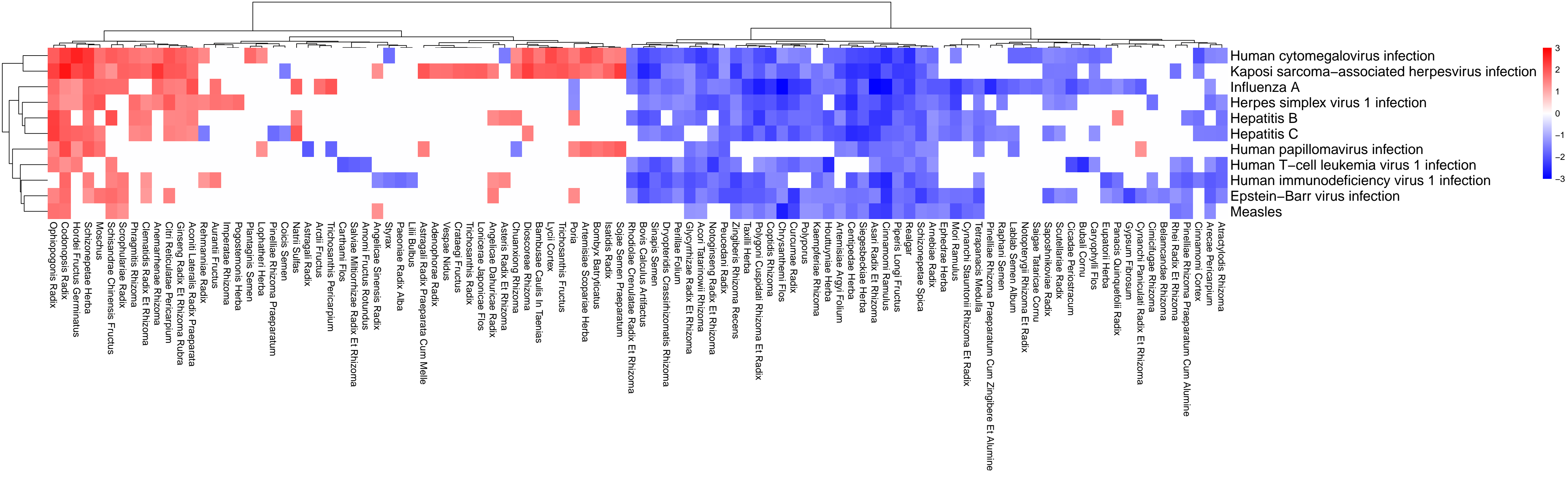

Supplement: Supplementary file 1 — Figure S1 [file 41392_2021_475_MOESM1_ESM.pdf]
