# Supplementary material for: Evaluation of the immunomodulatory effects of anti-COVID-19 TCM formulae by multiple virus-related pathways
Source: Signal Transduct Target Ther. 2021 Feb 4;6:50. doi: 10.1038/s41392-021-00475-w (PMC7860167; doi:10.1038/s41392-021-00475-w)
Supplement: Supplementary file 2 — Supplementary Files [file 41392_2021_475_MOESM2_ESM.pdf]

Supplementary Materials for

Evaluation of the immunomodulatory effects of anti-COVID-19 TCM formulae by multiple virus-related pathways

Liansheng Qiao [1,2,3†], Wenting Huang [1,2,3†], Xiaoling Zhang [4], Hongyan Guo [4], Dong Wang [5], Quansheng Feng [5], Ronghua Jin [6], Lan Xie [1,2,3,4*], Weimin Li [7*], Jing Cheng [1,2,3,4,5*]

Affiliations:

[1] State Key Laboratory of Membrane Biology, School of Medicine, Tsinghua University, Beijing 100084, China.

[2] Collaborative Innovation Center for Diagnosis and Treatment of Infectious Diseases, Hangzhou 310003, China.

[3] Medical Systems Biology Research Center, School of Medicine, Tsinghua University, Beijing 100084, China.

[4] National Engineering Research Center for Beijing Biochip Technology, Beijing 102206, China.

[5] School of Basic Medical Sciences, Chengdu University of Traditional Chinese Medicine, Chengdu 611137, China.

[6] Beijing Ditan Hospital, Capital Medical University, Beijing 100015, China.

[7] Department of Respiratory and Critic Care Medicine, West China Hospital, Sichuan University, Chengdu 610041, China.

[*]Correspondence to: Jing Cheng (jcheng@tsinghua.edu.cn); Lan Xie (xielan@tsinghua.edu.cn); Weimin Li (weimin003@163.com)

[†]These authors contributed equally: Liansheng Qiao, Wenting Huang

This PDF file includes:

Materials and Methods
Figure. S1
Captions for Data S1 to S5

Other Supplementary Materials for this manuscript include the following:

Data S1 to S5
Table S1. A set of 125 anti-COVID-19 TCM formulae was collected from public sources.
Table S2. The set of 196 TCMs constituting the 125 anti-COVID-19 TCM formulae.
Table S3. The list of 139 pathways and 3267 genes.
Table S4. The number of DEGs and NES for 166 TCMs on 11 virus-related pathways.
Table S5. The total pathway scores and NES for each pathway of the 125 formulae and 7 COVID-19-related samples.

Materials and Methods

Cell culture

THP-1 (National Infrastructure of Cell Line Resource) cells were cultured in RPMI1640 (HyClone, Logan, UT) supplemented with 10% fetal bovine serum and 1% penicillin-streptomycin (HyClone) at 37°C and 5% CO2 in a humidified atmosphere.

Probe design for high-throughput sequencing-based high-throughput screening (HTS^2)

A pair of probes was designed to hybridize with the 3' side and the 5' side of the transcript, which were designated the acceptor and donor. The acceptor comprises the common index primer and the 20-nt complementary sequence of the target. The donor comprises the specific 20-nt complementary sequence of the target and the P5 primer. Probes are preferentially designed against exon-exon junctions. Probes for a set of 3267 genes related to virus infection, immunity, inflammation, metabolism, cell proliferation, apoptosis, and migration were designed for HTS^2.

Construction of Traditional Chinese medicine (TCM) extract bank

A total of 166 TCMs from the 2015 Chinese Pharmacopeia were collected and enrolled in the TCM extract bank. TCMs were purchased from Anguo Changda Chinese Herbal Pieces Ltd. (Hebei, China) and Beijing Tongrentang Medicine Corporation Ltd. (Beijing, China). TCMs (10 g) were extracted and refluxed with 150 mL of 90% (v/v) ethanol for 3 hours and concentrated for 20 min using a Soxhlet apparatus (BUCHI Labortechnik AG, Flawil, Switzerland). All the extracts were further freeze dried and dissolved in dimethyl sulfoxide (DMSO) at a concentration of 50 mg/mL. There were some exceptions, *i.e.*, *Gypsum Fibrosum* (Shigao) (10 g) was immersed in 100 mL water and refluxed for 2 hours, the precipitate was obtained by centrifugation and subjected to the second reflux in 80 mL water for 1.5 hours followed by centrifugation. The supernatants collected from twice centrifugations were concentrated, freeze dried, and dissolved in RPMI1640 complete medium at a concentration of 200 μg/mL. *Borneolum Syntheticum* (Bingpian), *Styrax* (Suhexiang), *Moschus* (Shexiang), *Realgar* (Xionghuang) were directly smashed and dissolved in DMSO, and *Natrii Sulfas* (Mangxiao) and *Natrii Sulfas Exsiccatus* (Xuanmingfen) were directly dissolved in water.

HTS^2

THP-1 cells (4,000 per well) were seeded in 384-well plates (Corning Inc., Corning, NY) and incubated with 100 ng/mL phorbol-12-myristate-13-acetate (PMA) for 48 hours, followed by treatment with TCM crude extracts at 100 μg/mL or DMSO for another 24 hours. Cells were lysed and kept at −80°C until performing an RNA-mediated oligonucleotide annealing, selection, and ligation (RASL) assay. The RASL assay has been reported previously. Briefly, samples were heated at 65°C for 8 min to denature RNA. Then, the samples were incubated at 45°C for 60 min so that probes were annealed to total RNA and were paired with target mRNA. Biotinylated oligo (dT) and streptavidin-coated magnetic beads were used to capture mRNA so that the aligned probes could resist washing. After washing, the aligned probes were ligated by T4 ligase at 37°C for 60 min. During polymerase chain reaction (PCR) amplification, products from a single sample were indexed by limited PCR amplification with a set of barcode primers. The products were finally combined, purified, quantified, and subjected to high-throughput sequencing using the HiSeq Xten PE150 platform (Illumina, San Diego, CA). The sequencing data of HTS^2 were mapped with Bowtie2. Then, the value of $\log_2$ fold change ($\log_2$FC) of TCM (n=3) in comparison

with DMSO (n=16) after PMA treatment was normalized and calculated by DEseq2. The differentially expressed genes were identified with a FC $\geqslant$ 1.5 or FC $\leqslant$ 0.67 and p-value $\leqslant$ 0.05.

Pathway enrichment analysis

A gene set enrichment analysis (GSEA) was utilized to perform pathway enrichment analysis by using GSEA software 3.0 from the Broad Institute and University of California, San Diego. The GseaPreRanked tool (nperm=1,000, set_min=5, set_max=500, scoring_scheme=classic, random.seed=666) was used to analyze 3267 genes ranked by log_2FC after treatment. Significantly enriched pathways were determined with a cutoff of false discovery rate (FDR) < 0.25. The hierarchical clustering of pathways and samples was utilized to analyze the enriched pathway profiles of TCMs based on the normalized enrichment score (NES).

Transcriptome analysis of coronavirus infectious disease 2019 (COVID-19)-related samples

The transcriptome datasets of COVID-19-related samples were downloaded from Gene Expression Omnibus (GSE145926 and GSE147507). In GSE145926, leukocytes ($CD45^+$) and macrophages ($CD68^+$) in bronchoalveolar lavage fluid of six severe COVID-19 patients were collected together with those of three healthy controls. In GSE147507, Calu-3 cells, A549 cells, and normal human bronchial epithelial (NHBE) cells were infected with severe acute respiratory coronavirus 2 (SARS-CoV-2) at a multiplicity of infection (MOI) of 2 for 24 hours. In addition, A549 cells were infected with SARS-CoV-2 at a MOI of 0.2 for 24 hours. The lung tissues from postmortem COVID-19 patients were collected together with those of two healthy controls. The gene expression profiles were analyzed based on each group of COVID-19 samples compared with that of controls. The datasets mentioned above were designated COVID-19.Leukocytes, COVID-19.Macrophages, COVID-19.Calu3, COVID-19.A549.MOI2, COVID-19.NHBE, COVID-19.A549.MOI0.2, and COVID-19.Lung tissue, respectively, in this study.

Pathway simulation of the anti-COVID-19 formula

The NES of an anti-COVID-19 TCM formula for each pathway was simulated with the sum of the NES of each constitutive TCM from the formula for this pathway. The total pathway score of a formula was calculated by adding the NES of this formula on 11 virus-related pathways. Although 196 TCMs were included in the 125 formulae, 30 were excluded because of unfavorable accessibility or unsuccessful extraction. We computed the pathway score for each formula as follows:

$$\text{Pathway score} = \sum_{j=1}^{j} \sum_{i=1}^{i} NES_{ij}$$

where i is the number of TCMs contained in a formula and j is the number of pathways (j=11 in this study).

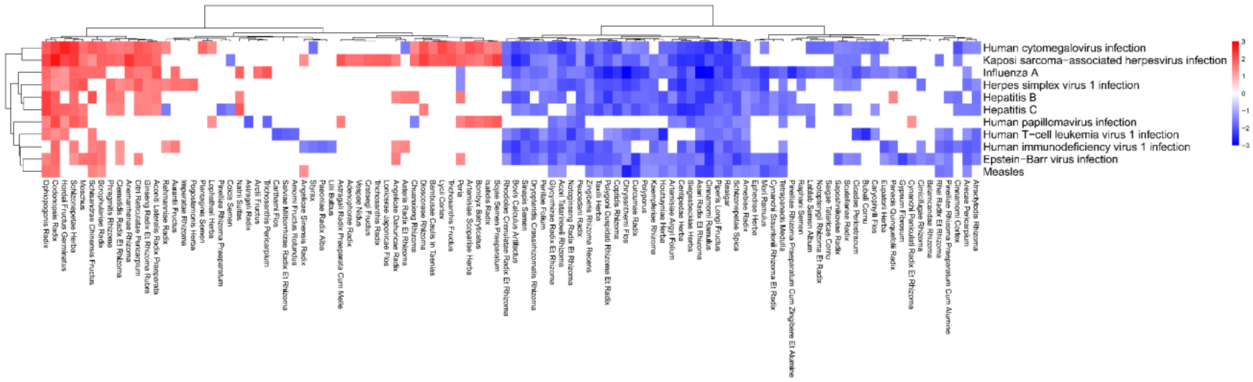

Figure. S1.

Heatmap of 11 virus-related pathways for 102 TCMs. The color of each spot in the heatmap represents the NES for each pathway in each sample (FDR < 0.25) along a color gradient from blue (NES < 0) to red (NES > 0).

Table S1. (separate file)

A set of 125 anti-COVID-19 TCM formulae was collected from public sources. The sources include the DTPC Versions 3, 4 and 6; other DTPCs from provinces and cities; the Handbook of Diagnosis and Treatment of the Pneumonia Caused by the Novel Coronavirus in TCM; Chinese medicine experts and hospital preparations. The constitutive TCMs for each formula are listed, together with the disease stage to which the formula is applicable.

Table S2. (separate file)

The set of 196 TCMs constituting the 125 anti-COVID-19 TCM formulae. The Chinese and Latin names of each TCM are listed, and the frequency of each TCM in the 125 formulae is recorded.

Table S3. (separate file)

The list of 139 pathways and 3267 genes. A set of 3267 genes are derived from 139 pathways related to virus infection, immunity, inflammation, metabolism, cell proliferation, apoptosis, and migration.

Table S4. (separate file)

The number of DEGs and NES for 166 TCMs on 11 virus-related pathways. The number of DEGs for 166 TCMs is counted, and the NES of 11 virus-related pathways for 166 TCMs is listed (FDR<0.25). The order of TCMs is according to their orders in the heatmap of Fig. S1.

Table S5. (separate file)

The total pathway scores and NES for each pathway of the 125 formulae and 7 COVID-19-related samples. The total pathway scores and NES for individual pathways for each formula or COVID-19-related sample are listed. The formula or sample is ordered as they appear in the heatmap of Fig. 1a.
